# Supplementary material for: Insights into Hunter syndrome from the structure of iduronate-2-sulfatase
Source: Nat Commun. 2017 Jun 8;8:15786. doi: 10.1038/ncomms15786 (PMC5472762; doi:10.1038/ncomms15786)
Supplement: Supplementary Information — Supplementary figures, supplementary table and supplementary references. [file ncomms15786-s1.pdf]

Supplementary Table 1. IDS missense mutations associated with Hunter syndrome

Mutation	ASA* (%)	B/E†	Phen.‡	Structural consequences	Ref.
L41P	0	B	M	β-strand m/c torsion angles incompatible with proline geometry	1
D45N	1.9	B		catalytic core disruption: loss of acidic s/c and stabilizing H-bonding network. Interference with Ca^{2+} ion coordination. Impaired enzyme activity	2,3
D45E					4
D45G					5
D45V					5
D46A	0.7	B		catalytic core disruption: loss of acidic s/c and stabilizing H-bonding network. Interference with Ca^{2+} ion coordination. Impaired enzyme activity	6
R48P	17.6		M	m/c geometry distortion and loss of large basic s/c together with stabilizing H-bonds in proximity to the active site	3,7,8
Y54D	4.6	B	S	large uncharged s/c to smaller acidic s/c: loss of stabilising H-bond interactions	9
S61P	0	B		m/c geometry distortion and loss of stabilising H-bond interactions	10
S61F				small polar to large polar or bulky hydrophobic s/c substitution: steric clash and loss of hydrogen bonds with neighbouring backbone amide of G52 and s/c carboxyl of D65	6
S61Y					5
N63D	2	B	M/I	minor conformation change, altered H-bonding by introduction of acidic s/c	9,11-14
N63K				impaired enzyme folding by introduction of large basic s/c and loss of H-bonding network	15
L67P	0	B		protein misfolding: disruption of alpha-helical structure by helix-breaking proline	3
A68E	6.6	B	S	significant conformational change caused by introduction of large acidic s/c into hydrophobic pocket	16
S71R	0	B	S	severe misfolding: introduction of large basic s/c into the hydrophobic pocket, loss of stabilizing interactions	17,18
S71N			M	steric clashes and loss of stabilizing interactions	19
L72P	0.8	B		m/c β-strand geometry distortion due to proline dihedral angle restraints	3
L73F	3.3	B	S	structural rearrangements due to introduction of planar s/c potentially involved in pi-stacking interaction	20
A77D	0	B		protein misfolding: destabilisation of central β-sheet by introduction of acidic s/c in tightly-packed hydrophobic core	21
A79E	0	B	M	small hydrophobic s/c to large acidic s/c: steric clashes near active site area	9
Q80R	1.1	B		protein misfolding: loss of multiple H-bonds between base of α-helix and β-strand at the interface between the two subdomains	6
Q80K					22
A82E	0	B		significant active site distortion by introduction of large acidic s/c	17
A82V				increased size of hydrophobic s/c: conformational changes in the tightly restrained active site area	23
C84W	-	-		loss of catalytic nucleophile precursor within CxPSR motif: failure of formylglycine generation and irreversible enzyme inactivation	6
C84Y					5
A85T	8.7	B	M/I/S	active-site conformational changes associated with hydrophobic s/c to polar or acidic s/c substitution	8,12,14,17,19,24
A85D					6
A85S			S		19
A85P				proline-induced m/c torsion angle changes in the active site area	25
P86R	1.3	B	S	severe catalytic core disruption: small s/c to large s/c within highly conserved CxPSR motif. Likely failure of FGE to modify C84 → FGly84	11,26,27
P86Q					12
P86L			I/S		8,19,28-30
S87N	0.8	B	M	CxPSR core motif modification by replacement with larger s/c residue. Catalytic core disruption and inefficient modification of C84 → FGly84 by FGE	28
R88H	0.6	B	I/S	loss of strictly conserved active site residue within CxPSR core motif. Charge neutralization in substrate-binding cleft. Disruption of the complex H-bonding network stabilizing catalytic core. Likely failure of FGE to modify C84 → FGly84	9,10,12,14,19,24,29,31
R88C			S		9,12,17,30,32
R88P			S		29,32
R88L			S		9
R88G			S		19
R88S					6
V89F	0.3	B		increased size of hydrophobic s/c , destabilisation or misfolding	19
L92P	0.3	B	S	proline-induced distortion of the α-helix adjacent to the active site	28

G94D	0	B	M	introduction of acidic s/c close to the active site, buried steric clash	26,27
R95G			I		11
R95T	0.5	B	M	loss of large basic s/c , disruption of complex H-bonding network stabilising loop	32
R95S					5
P97R	1.2	B		severe misfolding: introduction of large basic s/c at the two interface between the two subdomains	10
L102R	0.2	B	M	branched hydrophobic s/c to large basic s/c substitution near active site	9
Y108S	7.1	B	M	enzyme misfolding: large polar s/c to small polar/nonpolar s/c mutation resulting in loss of H-bond interaction stabilizing loop	3,19
Y108C			M		12
W109C	0	B		loss of large hydrophobic s/c together with stabilizing H-bond	33
W109R				severe misfolding: buried steric clash caused by large hydrophobic s/c to large basic s/c	6
R110S	9.2	B		loss of buried stabilising H-bond network by substitution to small polar s/c, local misfolding and displacement of glycan acceptor N115	4
N115Y	54.4	E		loss of glycosylation site affecting enzyme processing and trafficking	2
N115I					6
S117Y	14.8	B	S	loss of glycosylation at N115 due to corruption of N-X-S/T motif, bulky polar s/c introduced adjacent to glycosylation site	10,34
T118I	0	B	M/I/S	misfolding caused by loss of H-bond interaction stabilizing loop region	19,29,31
P120R	0	B	S	severe misfolding: introduction of large basic s/c and m/c torsion angle perturbation	26,27
P120H			M		26
Q121R	10.7	B	S	severe misfolding: replacement with large basic s/c accompanied by loss of m/c carbonyl group involved in H-bonding network	19
Q121H			S		20
E125V	60.2	E	M	solvent-exposed polar s/c to hydrophobic s/c substitution, loss of surface charge	12
T130I	0	B		polar s/c to nonpolar s/c substitution, loss of strong H-bond interaction	3
T130N			M	minor conformational change, polar s/c conserved. H-bonding may be preserved. Possible hyperglycosylation by introduction of N-X-S/T consensus sequence	35
S132W	0.9	B	S	active site disruption by introduction of large hydrophobic s/c near K135	20,36
G134R	0.6	B	S	misfolding and active site distortion: buried steric clash caused by large basic or acidic s/c in place of strictly conserved glycine	6,12
G134E					6
K135R	13.9	B	I	active site distortion: s/c substitution introduces guanidinium group adjacent to catalytic nucleophile	27,37
K135N			I	catalytic core disruption: loss of stabilising electrostatic interaction with the sulfate moiety. Impaired enzyme activity	28
H138D	24.5		M/I	catalytic core disruption: charge inversion or replacement with larger polar or basic s/c. Significant active site perturbation and loss of putative acid/base involved in proton transfer during sulfate elimination and FGly84 rehydration steps. Impaired enzyme activity	19
H138R					38
H138Y					5
G140V	30.4			protein misfolding: introduction of branched hydrophobic s/c in a solvent exposed area near active site	23
G140R				interference with substrate binding, steric hindrance by introduction of large basic s/c	39
S142F	0	B		active site distortion by replacement of small polar s/c with bulky polar or hydrophobic s/c, steric clash and loss of H-bond orienting H138	40
S142Y					41
S143F	1.1	B		small polar s/c to bulky hydrophobic s/c, steric clash and loss of stabilizing H-bond, possible displacement of H138	42,43
D148N	0	B	I	severe misfolding: loss of complex H-bond network stabilizing extended loop region	30
D148H					19
D148V					44
S152N	0	B		protein misfolding: disruption of strong stabilizing H-bond network	25
S152G					45
P157S	16.2			loss of correct m/c geometry in proline-rich coil, local destabilisation of extended loop region	41
H159P	45.1	E	S	loss of H-bond interaction, charge neutralisation and destabilization of putative substrate binding site	9
P160R	1	B		introduction of large basic s/c near active site, interference with catalysis and/or substrate binding	46
P160H					5
C171R	3.6	B		severe misfolding: loss of disulfide bond forming partner, buried steric clash	39
N181I	17.4		M	minor conformational changes resulting from polar s/c to nonpolar s/c substitution	32
L182P	0	B	I	loss of m/c hydrogen-bonding donor, destabilisation of flanking active site regions	8

**C184F**	0	B	M/I	severe misfolding: loss of disulfide bond forming partner and introduction of bulky hydrophobic s/c, buried steric clash	12
**C184W**					42
**D187V**	41.9	E		loop region destabilization by replacement with hydrophobic s/c at the solvent-exposed surface area, loss of stabilizing H-bond	47
**L196S**	0.3	B	M/I	minor conformational changes caused by hydrophobic s/c to polar s/c substitution	8,9
**P197R**	1.1	B		protein misfolding by introduction of large basic s/c and loss of proline-imposed structural rigidity of α-helix primary residue	10,18
**D198G**	0.1	B	M	loss of acidic s/c and stabilizing H-bond interactions	9
**D198N**					41
**A205P**	0	B	I	proline-induced m/c structural distortion of long α-helix flanking central β-sheet	11
**L221P**	0	B	I	proline-induced m/c geometry restraints, destabilisation of central β-sheet	26,27
**G224E**	0.1	B	S	active site disruption caused by introduction of large acidic s/c or hydrophobic s/c close to K135	9
**G224A**					44
**Y225D**	0	B	I	replacement with smaller acidic s/c leading to loss of stabilizing interactions	8
**K227M**			I		8
**K227Q**	1.6	B	S	misfolding caused by local charge inversion or loss of s/c ε-amino group participating in strong H-bonding	26
**K227E**					44
**P228L**					2,15
**P228T**	0	B	S	substitution of the turn-inducing proline preceding active site residue H229, protein misfolding and catalytic core disruption	14
**P228A**					10
**P228Q**					41
H229R			I/S		14,19
H229Y	30.7		S	catalytic core disruption: active site perturbation and loss of putative acid/base involved in proton transfer to the R-OH leaving group. Impaired enzyme activity	36
H229Q					6,47
**I230F**	2.7	B		active site distortion, introduction of bulky planar s/c causes steric clash and m/c displacement next to H229	6
**P231L**	2.2	B	M	active site distortion and local destabilisation , loss of the turn-inducing proline downstream from catalytic H229	14
**R233G**	0	B		protein misfolding: loss of guanidium group participating in complex H-bond network	48
**K236N**	44.7	E		surface charge alteration, loss of basic s/c, minor conformational change	22
**L259P**	0.4	B	S	proline-induced structural rigidity in the loop region	10,34
**Y264N**	0.3	B		protein misfolding caused by loss of loop-stabilizing H-bonding	49
**N265I**	0.5	B	I	substitution of polar s/c to hydrophobic or large basic s/c near K347, impaired enzyme activity	50
**N265K**					5,6
**P266R**	0.2	B		protein misfolding: introduction of large basic s/c at the interface between the two subdomains, destabilisation of extended loop region	2
**P266H**			M		29
**W267C**	0.2	B		loss of large s/c participating in noncovalent pi-stacking interactions	38
**D269V**	52.6	E		misfolding caused by loss of stabilizing H-bond interaction	30,42
**Q293H**	6.6	B	M	introduction of weakly basic s/c	16
**K295I**	35.0	E		loss of surface charge and steric clash with other buried branched hydrophobic s/c packing against central extended α-helix	41
**S299I**	1.7	B	M	central extended α-helix misfolding initiated by substitution of small polar s/c for branching hydrophobic s/c	34
**S303F**	0	B		small polar s/c to bulky hydrophobic s/c: loss of H-bond and steric clash destabilising central extended α-helix	6
**S305P**	0	B		severe misfolding due to m/c geometry alteration, helix-breaking proline substitution	51
**D308E**			M		14
**D308N**	0	B	I	protein misfolding: substitution of strictly conserved acidic s/c participating in strong H-bonding network	8
**D308Y**					52
**D308H**					5
**G312D**	0	B		severe misfolding: introduction of acidic s/c or free thiol group into tightly-packed hydrophobic core surrounding central extended α-helix	41
**G312C**					6
**L314P**	0	B	S	α-helix m/c disruption induced by helix-breaking proline substitution	8
**L314H**					5
**S333L**	0	B	S	small polar s/c to hydrophobic s/c substitution, disruption of extended H-bonding network stabilizing active site	3,7-10,12,19,37,46,53,54

D334G			S		53
D334N	4.1	B	M	catalytic core disruption: loss of acidic s/c and stabilizing H-bonding network. Interference with Ca^{2+} ion coordination. Impaired enzyme activity	19
D334Y					5
D334V					5
H335R	2.1	B	I	catalytic core disruption: loss of basic s/c or steric clash by introduction of large guanidinium group, interference with Ca^{2+} ion coordination. Impaired enzyme activity	10,19
H335P					6
G336R	0	B	S	active site distortion caused by introduction of large basic or acidic s/c close to the catalytic core, severe m/c displacement of H335	20
G336E			S		19
W337R	0.4	B	I	active site distortion, loss of hydrophobic core contacts and electrostatic disruption by introducing large basic s/c next to D46, interference with Ca^{2+} ion coordination	7,8
L339R	1.6	B	S	severe misfolding caused by steric clash following introduction of large basic s/c or proline-imposed backbone structural rigidity	19
L339P					55
G340D	0.7	B	M	destabilization of β-hairpin motif by introduction of acidic s/c	9
H342P	1	B		destabilization of β-hairpin motif by m/c geometry distortion or buried steric clash, loss of stabilising H-bond at interface between subdomains	5
H342Y			M		43
E341K	8.8	B	S	severe misfolding: acidic s/c to long basic s/c substitution resulting in local charge inversion and loss of strong H-bond interactions on either side of β-hairpin	20,43
E344K	0	B			5
W345C	2	B	M	loss of stabilizing pi-stacking interactions upstream of catalytic K347	28
W345R					41
A346D	5.4	B	M/S	active site perturbation by introduction of larger branched s/c adjacent to K347	54
A346V			M/S		56
K347I					12
K347Q	8.4	B	S	catalytic core disruption: loss of positive charge putatively involved in stabilising interactions with sulfate-ester bond of substrate. Impaired enzyme activity	20
K347T			S		13,57
K347E					15,47
Y348H	5.6	B		protein misfolding due to loss of the extended H-bonding network	42
S349I	4.3	B	S	misfolding caused by replacement of small polar s/c with hydrophobic branched s/c or large basic s/c	8,14,24
S349R					58
N350H	2.7	B		Protein misfolding: loss of stabilising H-bond at interface between two subdomains, steric clash following introduction of basic or bulky polar s/c	59
N350Y					6
P358R	0	B	S	protein misfolding: buried steric clash by introducing large basic s/c	36
L403R	1	B	I	misfolding and active site distortion: introduction of large basic s/c into the hydrophobic pocket near R88 and D334	12,19
L410P	0	B		proline-induced m/c distortion of α-helix flanking central β-sheet	60
C422Y					14,24
C422G	0.7	B	M	severe misfolding caused by loss of a disulfide bond-participating cysteine and buried steric clash by introduced s/c, destabilisation of extended loop region	27,37
C422R			S		32
P423S	50.4	E		loss of proline-imposed structural rigidity of extended loop and complex H-bonding network of arginine residue	3
C432Y	0.5	B	S	severe misfolding caused by loss of a disulfide bond participating cysteine stabilizing extended loop region	9
C432R					18
E434K	16.2			local charge inversion by substitution of acidic s/c to basic s/c, loss of strong H-bond required for stabilising two loop regions	2
S464R	1.7	B		severe misfolding: buried steric clash and loss of H-bond destabilising β-sheet at interface between the two subdomains	6
Q465P	8.3	B	S	severe misfolding: proline-induced m/c geometry distortion	49
P467L	0.1	B	S	severe misfolding: m/c geometry disturbance and buried steric clash by introduction of branched hydrophobic or large basic s/c	19,32
P467R					6
R468Q			I/S		3,7-9,11-13,17,19,20,31,32,34,61,62
R468L	0.8	B	M/I/S	severe misfolding: loss of buried guanidinium group of arginine participating in multiple strong H-bonds in core fold adjacent to the interface between the two subdomains. Possible destabilisation of active site K347	7,8,13
R468W			M/I/S		3,8,10,11,17,19,20,28,32,34,63
R468G			M/I/S		48
R468P					64

**P469H**	2.4	B	M	loop destabilization by loss of proline m/c geometry and replacement with large basic s/c	36
**P469R**					6
**D478Y**			S		9
**D478G**	6.8	B	M	protein misfolding caused by loss of the acidic s/c forming strong H-bonds required for stabilizing loop region stretching between two β strands	16
**D478N**					6
**P480Q**			M		
**P480L**	0.3	B	M	loop destabilization by introduction of branched or large basic s/c in a tightly packed hydrophobic pocket	19
**P480R**			S		
**I485R**	0	B	S	protein misfolding: substitution of branched hydrophobic s/c for large basic s/c, steric clash between adjacent loop regions	9,15,16
**I485K**					2, 10
**M488I**	0	B		protein misfolding: destabilisation of tightly-packed hydrophobic core at interface between the two subdomains. Severe buried clash by introduction of large basic s/c	65
**M488R**					6
**G489D**					15,38
**G489A**	0	B	I	protein misfolding: β-sheet destabilization by buried steric clashes between introduced s/c and closely packed neighbouring residues	65
**G489V**					6
**Y490S**	21.3		I	large polar s/c to smaller s/c substitution at the interface between the two subdomains resulting in loss of the stabilizing H-bond	19
**S491F**	0	B	M	protein misfolding by buried steric clash and loss of strong H-bond interactions stabilising β-sheet	34,43
**R493P**	6.5	B		protein misfolding: loss of multiple H-bonds stabilising interface between two subdomains, proline-imposed m/c geometry disruption of β-strand	6
**W502C**			S		2,6,14,24
**W502S**	0.3	B		severe misfolding due to loss of large hydrophobic s/c together with H-bond interaction stabilising β-sheet	46
**W502G**					6
**V503D**	0	B		introduction of acidic s/c, severe disruption of β-sheet hydrophobic core	5
**E521V**	3.8	B	S	severe β-sheet destabilization caused by loss of the acidic s/c participating in strong H-bonds formation	14,17,20,24
**E521K**			S		20
**L522P**	0.9	B		replacement with β-sheet-breaking proline residue	10,47,50
**Y523C**	1.8	B	M	β-sheet destabilization caused by loss of H-bond interaction	36

Overall **212** point mutations of **123** residues. Note that 5 mutations reported to be associated with Hunter syndrome have been omitted on the basis that they are observed at too high frequency in the Exome Aggregation Consortium database[66]: T214M[41], frequency=0.005060; D252N[12], 0.002465; P261A[10], 0.0002623; T309A[14], 0.001460; R313C[14], 0.0001141

[*]ASA: accessible surface area: percentage of surface area of each residue that is accessible to solvent calculated with the program GetArea.
[†]B/E: buried/exposed: residues are considered to be buried if ASA is less than 10% and to be solvent exposed if the ASA value exceeds 40%.
[‡]Phen: Hunter syndrome disease phenotype (if reported) as mild (M), intermediate (I) or severe (S)
Abbreviations: H-bond, hydrogen-bond; s/c, side chain; m/c, main chain. Residues conserved among other Human sulfatases are underlined and IDS active site residues are italicized. Multiple mutations of the same residue are grouped by colour.

Total: **542** mutation entries, HGMD (http://www.hgmd.cf.ac.uk/ac/index.php) June 2015

Missense/nonsense: **280** entries
Splicing: **50** entries
Regulatory: **0** entries
Small deletions: **99** entries
Small insertions: **43** entries
Small indels: **11** entries (deleted/inserted bases)
Gross deletions: **40** entries
Gross insertions: **4** entries
Complex rearrangements: **15** entries
Repeat variations: **0** entries

Supplementary Fig. 1

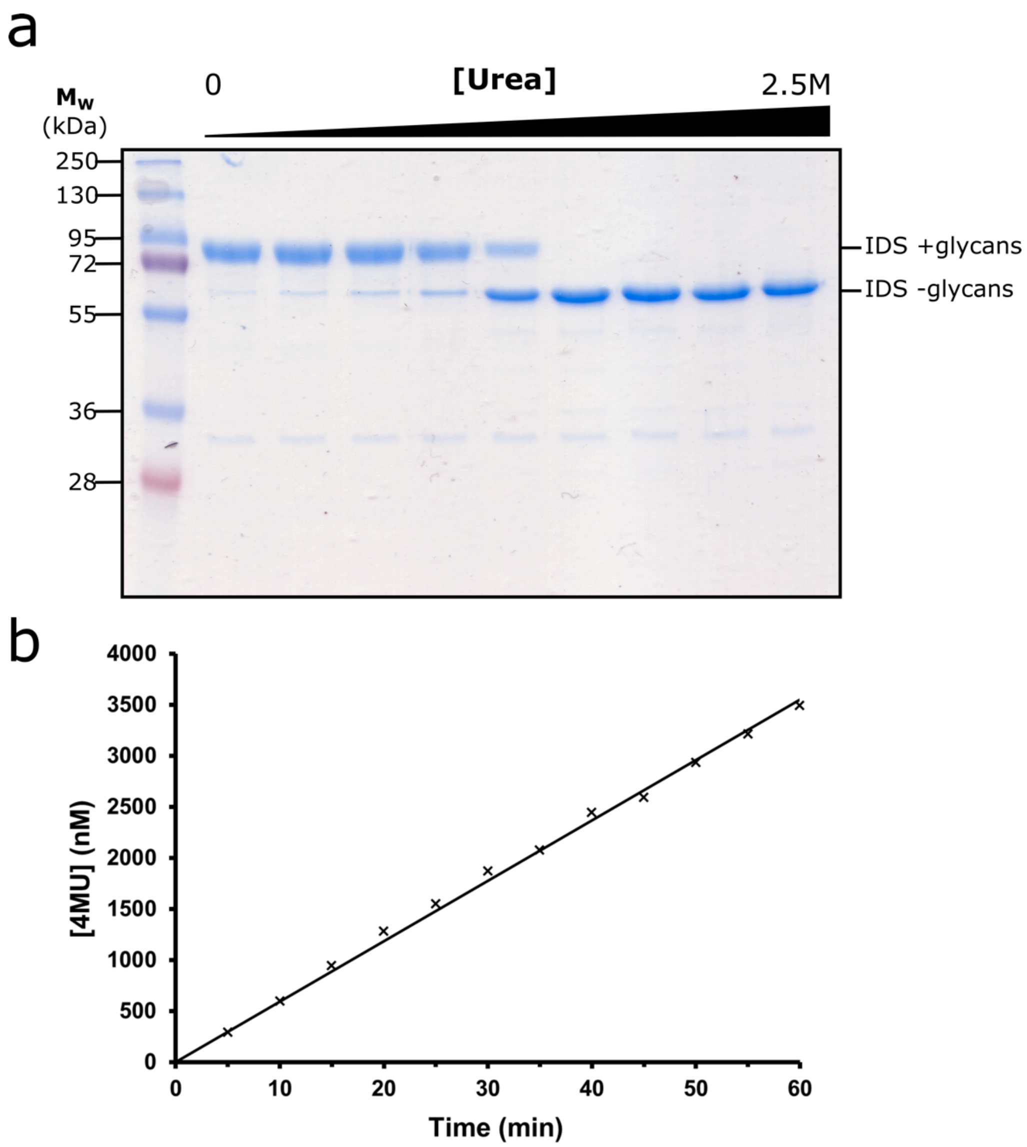

Supplementary Fig. 1. Biochemical properties of IDS protein. (**a**) SDS-PAGE analysis of IDS protein deglycosylated with PNGase F (5 hr, 310 K) following partial denaturation with increasing concentrations of urea (0, 0.05, 0.1, 0.2, 0.5, 1.0, 1.5, 2.0, 2.5M). (**b**) IDS activity plot showing linear increase in product 4-methylumbelliferone (4MU) with time. Assay was performed with 13.2 nM glycosylated IDS, 0.3 mM substrate 4-methylumbelliferyl-α-L-iduronide-2-sulfate (MU-αIdoA-2S) at pH 5.0, room temperature (293 K).

Supplementary Fig. 2

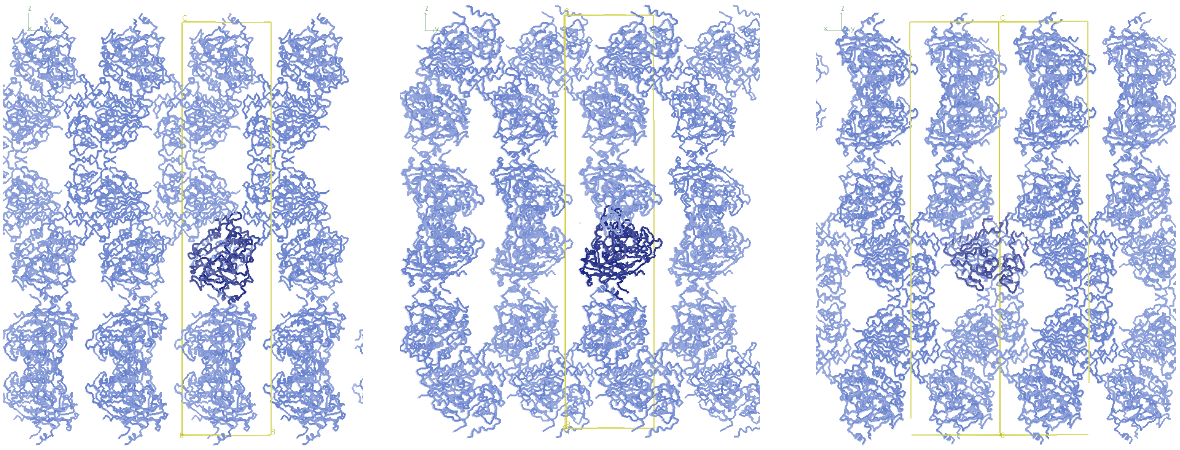

Supplementary Fig. 2. Crystal packing. Three views of the crystal packing are shown, rotated by 120 degrees around the vertical (*c*) axis. The unique molecule is shown in dark blue, with symmetry-related copies in light blue, and the unit cell is outlined in yellow. Extensive contacts form filaments of molecules along the vertical axis of the crystal, but the filaments only contact each other twice per unit cell in each of the horizontal (*a*, *b*, *a*+*b*) directions.

Supplementary Fig. 3

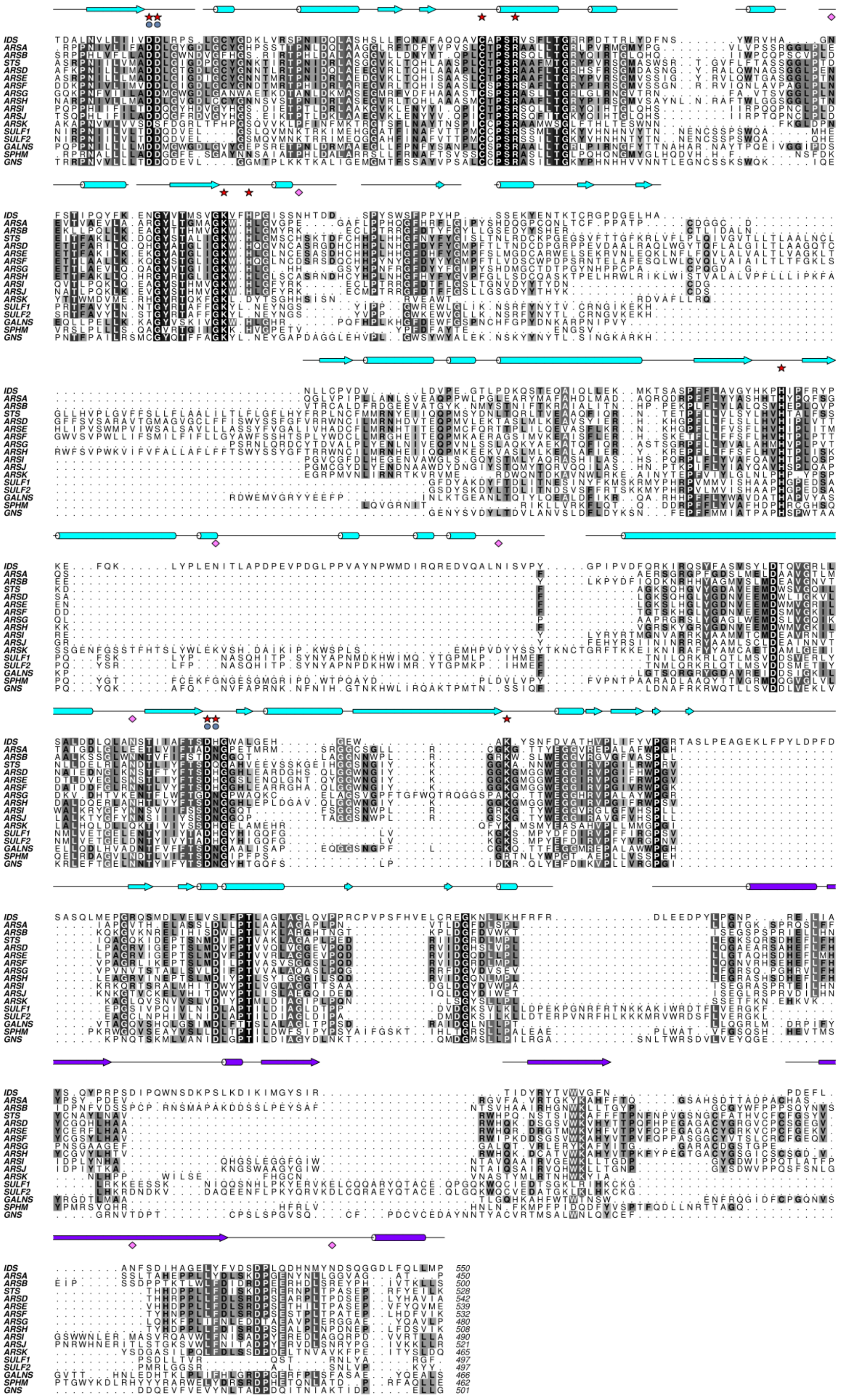

Supplementary Fig. 3. Amino acid sequence alignment of human sulfatases. Regions of sequence identity are highlighted, gaps are indicated by dots. The IDS secondary structure is superimposed for reference, with SD1 coloured in light blue and SD2 in purple. Active site residues (red stars), metal binding residues (grey circles) and N-linked glycosylation sites (pink diamonds) are shown. The aligned sequences are truncated before the IDS N-terminus and after the IDS C-terminus.

Supplementary Fig. 4

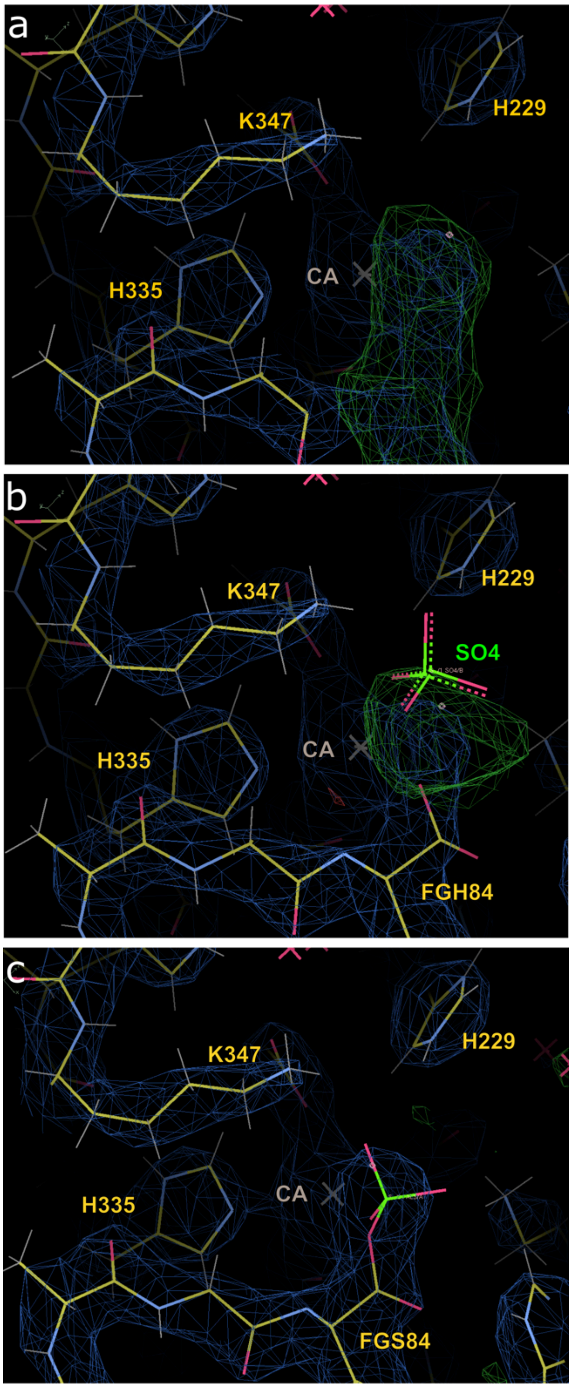

Supplementary Fig. 4. Refinement of the covalently-modified FGS84 residue. $2mF_O$-DF_C maps are shown in blue, contoured at 0.38 $e^- Å^{-3}$; mF_O-DF_C difference maps are shown in red and green, contoured at ± 3.0 times the rms value of the map. (a) Maps prior to modelling FGly84 and sulfate moiety. (b) Maps following refinement of FGH84 residue with a non-covalently bound sulfate ion as observed in the high-resolution PAS structure, oriented and restrained with reference to the high-resolution PAS structure [67]. (c) Maps following refinement of FGS84, with covalently-bound sulfate. The sulfate moiety was defined as a separate occupancy group.

Supplementary Fig. 5

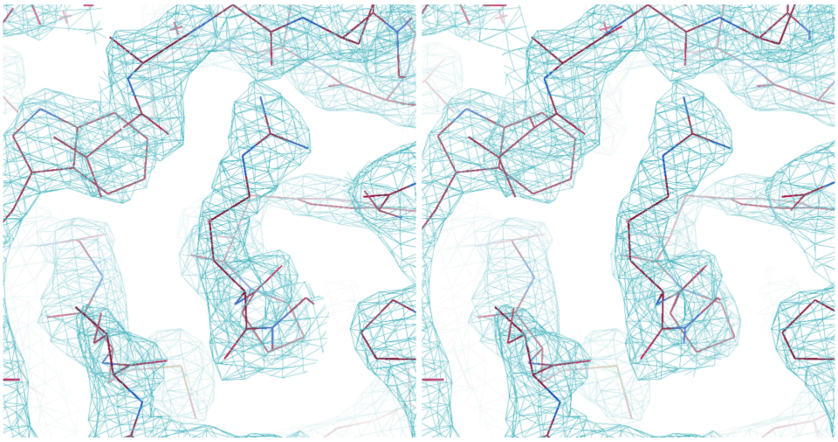

Supplementary Fig. 5. Stereo image of Arg468 in electron density. The environment is shown for Arg468, which has been identified in a number of mutations associated with Hunter syndrome cases. The $2mF_O$-DF_C electron density map, shown in cyan, is contoured at 0.28 $e^- \text{Å}^{-3}$.

Supplementary Fig. 6

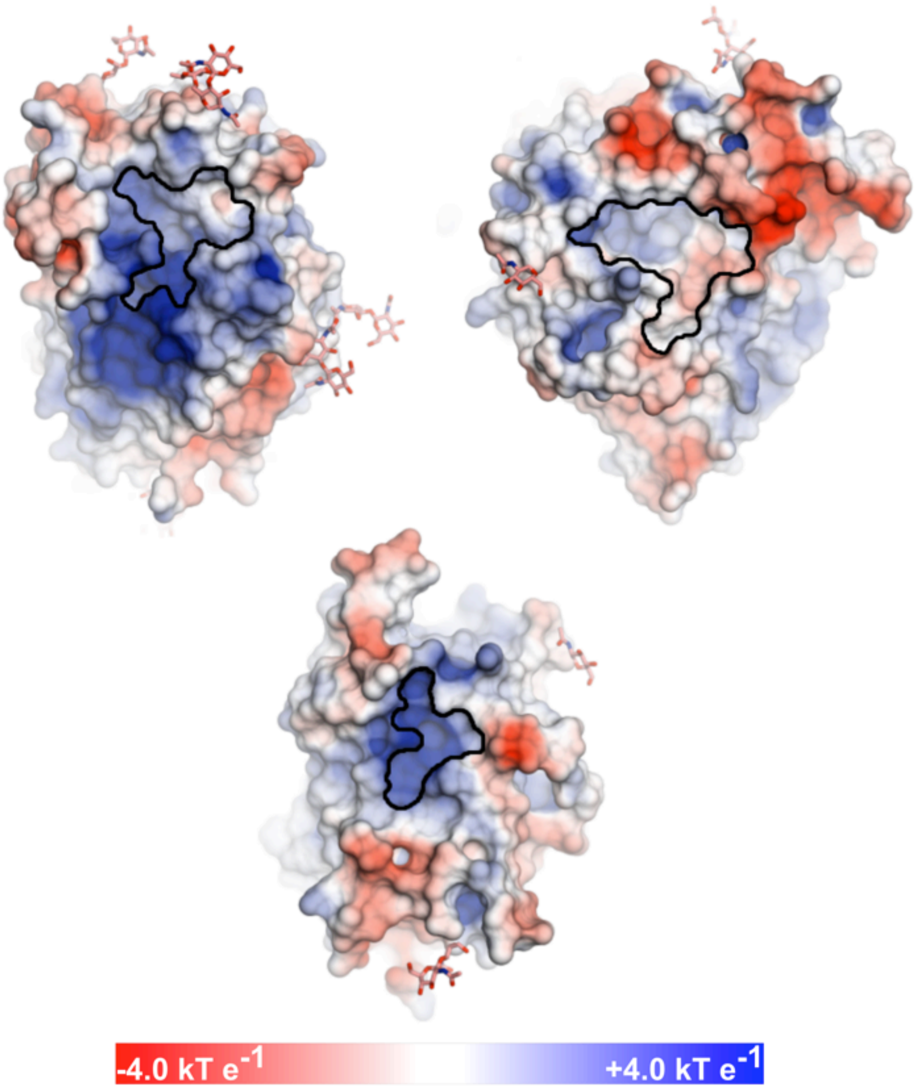

Supplementary Fig. 6. IDS surface cavities away from the active site. Showing three orthogonal views of IDS surface cavities deeper than four solvent radii. The IDS surface is coloured by electrostatic potential at the solvent-accessible surface from red (negative, -4.0 kT e^{-1}) to blue (positive, +4.0 kT e^{-1}). Electrostatic potential was calculated using a pH value of 4.8 when assigning side-chain protonation.

Supplementary References

1. Cudry, S. *et al.* MPS II in females: molecular basis of two different cases. *J Med Genet* **37**, E29 (2000).
2. Vafiadaki, E. *et al.* Mutation analysis in 57 unrelated patients with MPS II (Hunter's disease). *Arch Dis Child* **79**, 237-241 (1998).
3. Zhang, H. *et al.* Analysis of the IDS gene in 38 patients with Hunter syndrome: the c.879G>A (p.Gln293Gln) synonymous variation in a female create exonic splicing. *PLoS One* **6**, e22951 (2011).
4. Guillen-Navarro, E. *et al.* Clinical manifestations in female carriers of mucopolysaccharidosis type II: a Spanish cross-sectional study. *Orphanet J Rare Dis* **8**, 92 (2013).
5. Brusius-Facchin, A. C. *et al.* Mucopolysaccharidosis type II: identification of 30 novel mutations among Latin American patients. *Mol Genet Metab* **111**, 133-138 (2014).
6. Pollard, L. M., Jones, J. R. & Wood, T. C. Molecular characterization of 355 mucopolysaccharidosis patients reveals 104 novel mutations. *J Inherit Metab Dis* **36**, 179-187 (2013).
7. Sukegawa, K. *et al.* Mucopolysaccharidosis type II (Hunter disease): identification and characterization of eight point mutations in the iduronate-2-sulfatase gene in Japanese patients. *Hum Mutat* **6**, 136-143 (1995).
8. Isogai, K. *et al.* Mutation analysis in the iduronate-2-sulphatase gene in 43 Japanese patients with mucopolysaccharidosis type II (Hunter disease). *J Inherit Metab Dis* **21**, 60-70 (1998).
9. Karsten, S. *et al.* Mutational spectrum of the iduronate-2-sulfatase (IDS) gene in 36 unrelated Russian MPS II patients. *Hum Genet* **103**, 732-735 (1998).
10. Sohn, Y. B. *et al.* Identification of 11 novel mutations in 49 Korean patients with Mucopolysaccharidosis Type II. *Clin Genet* (2011).
11. Goldenfum, S. L., Young, E., Michelakakis, H., Tsagarakis, S. & Winchester, B. Mutation analysis in 20 patients with Hunter disease. *Hum Mutat* **7**, 76-78 (1996).
12. Rathmann, M. *et al.* Mucopolysaccharidosis type II (Hunter syndrome): mutation "hot spots" in the iduronate-2-sulfatase gene. *Am J Hum Genet* **59**, 1202-1209 (1996).
13. Villani, G. R. *et al.* Mucopolysaccharidosis type II: identification of six novel mutations in Italian patients. *Hum Mutat* **10**, 71-75 (1997).
14. Gort, L., Chabas, A. & Coll, M. J. Hunter disease in the Spanish population: molecular analysis in 31 families. *J Inherit Metab Dis* **21**, 655-661 (1998).
15. Lin, S. P. *et al.* Detection of Hunter syndrome (mucopolysaccharidosis type II) in Taiwanese: biochemical and linkage studies of the iduronate-2-sulfatase gene defects in MPS II patients and carriers. *Clin Chim Acta* **369**, 29-34 (2006).
16. Schroder, W., Wulff, K., Wehnert, M., Seidlitz, G. & Herrmann, F. H. Mutations of the iduronate-2-sulfatase (IDS) gene in patients with Hunter syndrome (mucopolysaccharidosis II). *Hum Mutat* **4**, 128-131 (1994).
17. Li, P., Bellows, A. B. & Thompson, J. N. Molecular basis of iduronate-2-sulphatase gene mutations in patients with mucopolysaccharidosis type II (Hunter syndrome). *J Med Genet* **36**, 21-27 (1999).
18. Lualdi, S. *et al.* Identification of nine new IDS alleles in mucopolysaccharidosis II. Quantitative evaluation by real-time RT-PCR of mRNAs sensitive to nonsense-mediated and nonstop decay mechanisms. *Biochim Biophys Acta* **1762**, 478-484 (2006).
19. Froissart, R. *et al.* Identification of iduronate sulfatase gene alterations in 70 unrelated Hunter patients. *Clin Genet* **53**, 362-368 (1998).
20. Lissens, W., Seneca, S. & Liebaers, I. Molecular analysis in 23 Hunter disease families. *J Inherit Metab Dis* **20**, 453-456 (1997).

21 Grinberg, H. *et al.* The first cardiac transplant experience in a patient with mucopolysaccharidosis. *Cardiovasc Pathol* **21**, 358-360 (2012).
22 Gucev, Z. S. *et al.* Hunter syndrome (Muccopolysaccharridosis Type II) in Macedonia and Bulgaria. *Prilozi* **32**, 187-198 (2011).
23 Lualdi, S. *et al.* Multiple cryptic splice sites can be activated by IDS point mutations generating misspliced transcripts. *J Mol Med (Berl)* **84**, 692-700 (2006).
24 Gort, L., Coll, M. J. & Chabas, A. Mutations in the iduronate-2-sulfatase gene in 12 Spanish patients with Hunter disease. *Hum Mutat* **Suppl 1**, S66-68 (1998).
25 Cooper, A., Whitehead, K. L. & Wraith, J. E. Human Gene Mutations. *Hum Genet* **108**, 1-84 (2001).
26 Hopwood, J. J. *et al.* Molecular basis of mucopolysaccharidosis type II: mutations in the iduronate-2-sulphatase gene. *Hum Mutat* **2**, 435-442 (1993).
27 Bunge, S. *et al.* Iduronate-2-sulfatase gene mutations in 16 patients with mucopolysaccharidosis type II (Hunter syndrome). *Hum Mol Genet* **2**, 1871-1875 (1993).
28 Popowska, E. *et al.* Mutations of the iduronate-2-sulfatase gene in 12 Polish patients with mucopolysaccharidosis type II (Hunter syndrome). *Hum Mutat* **5**, 97-100 (1995).
29 Balzano, N., Villani, G. R., Grosso, M., Izzo, P. & Di Natale, P. Detection of four novel mutations in the iduronate-2-sulfatase gene. Mutations in brief no. 123. Online. *Hum Mutat* **11**, 333 (1998).
30 Alves, S. *et al.* Molecular characterization of Portuguese patients with mucopolysaccharidosis type II shows evidence that the IDS gene is prone to splicing mutations. *J Inherit Metab Dis* **29**, 743-754 (2006).
31 Villani, G. R., Daniele, A., Balzano, N. & Di Natale, P. Expression of five iduronate-2-sulfatase site-directed mutations. *Biochim Biophys Acta* **1501**, 71-80 (2000).
32 Moreira da Silva, I. *et al.* Molecular basis of mucopolysaccharidosis type II in Portugal: identification of four novel mutations. *Clin Genet* **60**, 316-318 (2001).
33 Emre, S. *et al.* Biochemical and molecular analysis of mucopolysaccharidoses in Turkey. *Turk J Pediatr* **44**, 13-17 (2002).
34 Kim, C. H. *et al.* Mutational spectrum of the iduronate 2 sulfatase gene in 25 unrelated Korean Hunter syndrome patients: identification of 13 novel mutations. *Hum Mutat* **21**, 449-450 (2003).
35 Boyadjiev, S., Bivina, L., McGinniss, M. & Hata, A. Very mild clinical manifestation of Hunter syndrome due to a novel IDS mutation (vol 105, pg S15, 2012). *Molecular Genetics and Metabolism* **106**, 255-255 (2012).
36 Jonsson, J. J., Aronovich, E. L., Braun, S. E. & Whitley, C. B. Molecular diagnosis of mucopolysaccharidosis type II (Hunter syndrome) by automated sequencing and computer-assisted interpretation: toward mutation mapping of the iduronate-2-sulfatase gene. *Am J Hum Genet* **56**, 597-607 (1995).
37 Bunge, S. *et al.* Mutation analysis of the iduronate-2-sulfatase gene in patients with mucopolysaccharidosis type II (Hunter syndrome). *Hum Mol Genet* **1**, 335-339 (1992).
38 Chang, J. H. *et al.* Expression studies of mutations underlying Taiwanese Hunter syndrome (mucopolysaccharidosis type II). *Hum Genet* **116**, 160-166 (2005).
39 Kato, T. *et al.* Mutational and structural analysis of Japanese patients with mucopolysaccharidosis type II. *J Hum Genet* **50**, 395-402 (2005).
40 Fernandez-Marmiesse, A. *et al.* Assessment of a targeted resequencing assay as a support tool in the diagnosis of lysosomal storage disorders. *Orphanet J Rare Dis* **9**, 59 (2014).
41 Amartino, H. *et al.* Identification of 17 novel mutations in 40 Argentinean unrelated families with mucopolysaccharidosis type II (Hunter syndrome). *Molecular Genetics and Metabolism Reports* **1**, 401-406 (2014).

42 Karsten, S. L. *et al.* Identification of 9 novel IDS gene mutations in 19 unrelated Hunter syndrome (mucopolysaccharidosis Type II) patients. Mutations in brief no. 202. Online. *Hum Mutat* **12**, 433 (1998).
43 Vallance, H. D. *et al.* Identification of 6 new mutations in the iduronate sulfatase gene. Mutation in brief no. 233. Online. *Hum Mutat* **13**, 338 (1999).
44 Keeratichamroen, S. *et al.* Molecular analysis of the iduronate-2-sulfatase gene in Thai patients with Hunter syndrome. *J Inherit Metab Dis* **31 Suppl 2**, S303-311 (2008).
45 Tchan, M. C., Devine, K. T. & Sillence, D. O. Three Adult Siblings with Mucopolysaccharidosis Type II (Hunter Syndrome): A Report on Clinical Heterogeneity and 12 Months of Therapy with Idursulfase. *JIMD Rep* **1**, 57-64 (2011).
46 Flomen, R. H., Green, P. M., Bentley, D. R., Giannelli, F. & Green, E. P. Detection of point mutations and a gross deletion in six Hunter syndrome patients. *Genomics* **13**, 543-550 (1992).
47 Whitehead, K. L., Cooper, A. & Wraith, J. E. Human Gene Mutations. *Hum Genet* **108**, 1-84 (2001).
48 Chistiakov, D. A. *et al.* Genetic analysis of 17 children with Hunter syndrome: identification and functional characterization of four novel mutations in the iduronate-2-sulfatase gene. *J Genet Genomics* **41**, 197-203 (2014).
49 Hartog, C., Fryer, A. & Upadhyaya, M. Mutation analysis of iduronate-2-sulphatase gene in 24 patients with Hunter syndrome: characterisation of 6 novel mutations. Mutation in brief no. 249. Online. *Hum Mutat* **14**, 87 (1999).
50 Filocamo, M. *et al.* Molecular analysis of 40 Italian patients with mucopolysaccharidosis type II: New mutations in the iduronate-2-sulfatase (IDS) gene. *Hum Mutat* **18**, 164-165 (2001).
51 Chang, J. H., Lee-Chen, G. J., Lin, S. P. & Chuang, C. K. Characterization of a novel p.S305P and a known c.1006+5G>C splice site mutation in human iduronate-2-sulfatase associated with mucopolysaccharidosis type II. *Clin Chim Acta* **384**, 167-170 (2007).
52 Zhang, C. Y., Li, L. Y., Liu, S. F., Fu, J. J. & Lu, G. X. [Detection of a new mutation (G1253T) of iduronate-2-sulfatase gene for the patient with mucopolysaccharidosis type II]. *Zhonghua Yi Xue Yi Chuan Xue Za Zhi* **21**, 269-271 (2004).
53 Li, P. & Thompson, J. N. Detection of four novel mutations in the iduronate-2-sulphatase gene by single-strand conformation polymorphism analysis of genomic amplicons. *J Inherit Metab Dis* **19**, 93-94 (1996).
54 Olsen, T. C. *et al.* Mutations in the iduronate-2-sulfatase gene in five Norwegians with Hunter syndrome. *Hum Genet* **97**, 198-203 (1996).
55 Guo, Y. B. & Du, C. S. [Detection of a new mutation (T1140C)in a Chinese Guangdong patient with hunter syndrome]. *Yi Chuan* **28**, 521-524 (2006).
56 Li, P., Huffman, P. & Thompson, J. N. Mutations of the iduronate-2-sulfatase gene on a T146T background in three patients with Hunter syndrome. *Hum Mutat* **5**, 272-274 (1995).
57 Bonuccelli, G. *et al.* The effect of four mutations on the expression of iduronate-2-sulfatase in mucopolysaccharidosis type II. *Biochim Biophys Acta* **1537**, 233-238 (2001).
58 Cobos, P. N., Steglich, C., Santer, R., Lukacs, Z. & Gal, A. Dried blood spots allow targeted screening to diagnose mucopolysaccharidosis and mucolipidosis. *JIMD Rep* **15**, 123-132 (2015).
59 Piotrowska, E. *et al.* Correlation between severity of mucopolysaccharidoses and combination of the residual enzyme activity and efficiency of glycosaminoglycan synthesis. *Acta Paediatr* **98**, 743-749 (2009).

60 Ben Simon-Schiff, E., Bach, G., Hopwood, J. J. & Abeliovich, D. Mutation analysis of Jewish Hunter patients in Israel. *Hum Mutat* **4**, 263-270 (1994).
61 Whitley, C. B. *et al.* Caveat to genotype-phenotype correlation in mucopolysaccharidosis type II: discordant clinical severity of R468W and R468Q mutations of the iduronate-2-sulfatase gene. *Hum Mutat* **2**, 235-237 (1993).
62 Sukegawa, K. *et al.* Hunter disease in a girl caused by R468Q mutation in the iduronate-2-sulfatase gene and skewed inactivation of the X chromosome carrying the normal allele. *Hum Mutat* **10**, 361-367 (1997).
63 Crotty, P. L., Braun, S. E., Anderson, R. A. & Whitley, C. B. Mutation R468W of the iduronate-2-sulfatase gene in mild Hunter syndrome (mucopolysaccharidosis type II) confirmed by in vitro mutagenesis and expression. *Hum Mol Genet* **1**, 755-757 (1992).
64 Charoenwattanasatien, R. *et al.* Decreasing activity and altered protein processing of human iduronate-2-sulfatase mutations demonstrated by expression in COS7 cells. *Biochem Genet* **50**, 990-997 (2012).
65 Ricci, V. *et al.* Expression studies of two novel in CIS-mutations identified in an intermediate case of Hunter syndrome. *Am J Med Genet A* **120A**, 84-87 (2003).
66 Lek, M. *et al.* Analysis of protein-coding genetic variation in 60,706 humans. *Nature* **536**, 285-291 (2016).
67 Boltes, I. *et al.* 1.3 A structure of arylsulfatase from Pseudomonas aeruginosa establishes the catalytic mechanism of sulfate ester cleavage in the sulfatase family. *Structure* **9**, 483-491 (2001).
